# The Role of Umbilical Cord Mesenchymal Stem Cell-Derived Extracellular Vesicles in Modulating Dermal Fibroblast Activity: A Pathway to Enhanced Tissue Regeneration

**DOI:** 10.3390/biology14020150

**Published:** 2025-02-01

**Authors:** Muttiah Barathan, Kow Jack Ham, Hui Yin Wong, Jia Xian Law

**Affiliations:** 1 Department of Tissue Engineering and Regenerative Medicine, Faculty of Medicine, Universiti Kebangsaan Malaysia, Cheras, Kuala Lumpur 56000, Malaysia; barathanmuttiah@ukm.edu.my; 2 Humanrace Sdn. Bhd., 8-5, Setia Avenue, Jalan Setia Prima (S) U13/S, Setia Alam, Seksyen 13, Shah Alam 40170, Selangor, Malaysia; ham@nexus-scientific.com (K.J.H.); rosiewong@nexus-scientific.com (H.Y.W.); 3 Nexus Scientific Sdn. Bhd., 8-5, Setia Avenue, Jalan Setia Prima (S) U13/S, Setia Alam, Seksyen 13, Shah Alam 40170, Selangor, Malaysia

**Keywords:** dermal fibroblast, extracellular vesicle, mesenchymal stem cell, umbilical cord, wound healing

## Abstract

Extracellular vesicles (EVs) secreted by umbilical cord-derived mesenchymal stem cells (UC-MSCs) hold significant promise as therapeutic agents in regenerative medicine. This study investigates the effects of UC-MSC-derived EVs on dermal fibroblast function, and their potential in wound healing applications. EVs were characterized by nanoparticle tracking analysis and transmission electron microscopy, revealing a mean size of 118.6 nm, consistent with exosomal properties. Dermal fibroblasts were treated with varying concentrations of EVs (25–100 µg/mL), and their impacts on cellular metabolism, mitochondrial activity, reactive oxygen species (ROS) production, wound closure, inflammatory cytokine secretion, growth factor production, and extracellular matrix (ECM) gene expression were evaluated. At lower concentrations (25–50 µg/mL), EVs significantly enhanced fibroblast metabolic and mitochondrial activity. However, higher concentrations (≥75 µg/mL) increased ROS levels, suggesting potential hormetic effects. EVs also modulated inflammation by reducing pro-inflammatory cytokines (IL-6, TNF-α) while promoting pro-regenerative cytokines (IL-33, TGF-β). Treatment with 50 µg/mL of EVs optimally stimulated wound closure and growth factor secretion (VEGF, BDNF, KGF, IGF), and upregulated ECM-related gene expression (type I and III collagen, fibronectin). These findings demonstrate that UC-MSC-derived EVs exert multifaceted effects on dermal fibroblast function, including enhanced cellular energetics, stimulation of cell migration, regulation of inflammation, promotion of growth factor production, and increased ECM synthesis. This study highlights the potential of EVs as a novel therapeutic strategy for wound healing and tissue regeneration, emphasizing the importance of optimizing EV concentration for maximal therapeutic efficacy.

## Simple Summary

Wound healing is a vital process for restoring skin integrity after an injury, and scientists are actively exploring new innovative approaches to enhance this process. This study examines the use of tiny particles known as extracellular vesicles (EVs), secreted by umbilical cord-derived mesenchymal stem cells (UC-MSCs), in promoting wound repair. EVs have been shown to support skin cell migration and proliferation, both of which are essential for effective healing. The objective of this research was to investigate how EVs influence the function of dermal fibroblasts, a key type of skin cell involved in wound repair, and to determine their role in enhancing the healing process. The results indicate that EVs can increase the activity of dermal fibroblasts and stimulate the production of important proteins that promote healing. Additionally, they help to reduce inflammation, which can otherwise impede recovery. The study also identified the optimal amount of EVs that maximizes healing speed by boosting the production of growth factors and supporting the formation of new skin tissue. The findings of this research suggest that EVs derived from UC-MSCs hold great promise as a novel therapeutic option for wound care. They could be particularly beneficial for individuals with slow-healing wounds, such as the elderly or those suffering from chronic conditions.

## 1. Introduction

Wound healing is a complex biological process involving the intricate interplay of various cellular components, signaling pathways, and extracellular matrix (ECM) elements [1]. It progresses through four distinct stages: hemostasis, inflammation, proliferation, and remodeling. During the initial stages, immune cells clear debris and release cytokines, while fibroblasts and endothelial cells contribute to collagen production and angiogenesis, both of which are essential for tissue repair [2]. Keratinocytes play a crucial role in re-epithelialization, restoring the skin’s barrier function. The ECM, composed of proteins like collagen and fibronectin, provides structural support and biochemical signals that are critical for cell migration and proliferation [3,4]. Disruptions to this process can result in impaired healing, highlighting the importance of new therapeutic strategies, such as mesenchymal stem cell-derived extracellular vesicles (MSC-EVs), in enhancing tissue repair [5,6].

EVs, which include exosomes and microvesicles, are membrane-bound structures that mediate intercellular communication by transferring proteins, nucleic acids, lipids, and other bioactive molecules [7,8]. Their cargo plays a pivotal role in biological processes such as epithelial–mesenchymal transition (EMT) and ECM synthesis, both of which are vital for effective wound healing [9,10]. The therapeutic properties of MSC-EVs are largely attributed to their ability to modulate paracrine signaling pathways. These pathways involve the release of regulatory factors, chemokines, cytokines, growth factors, and nucleic acids, collectively contributing to the healing process [11,12].

The composition of EVs varies based on their cellular origin, influencing their functional properties. MSC-EVs, in particular, are renowned for their regenerative potential, due to their unique mixture of bioactive molecules that promote tissue repair and reduce scar formation [13,14]. These vesicles enhance the proliferation and migration of dermal fibroblasts (DFs) and increase the angiogenic potential of dermal endothelial cells, underscoring their role in accelerating wound healing [15,16]. The interaction between EVs and the ECM is critical for wound healing, since EVs not only deliver signaling molecules that regulate ECM remodeling, but also interact directly with ECM components to influence cell behavior [17,18]. This interaction is especially significant, because the ECM provides both structural and biochemical support to surrounding cells, facilitating cellular communication during tissue repair [19]. Understanding how EVs modulate the synthesis and composition of the ECM could lead to more targeted therapeutic strategies in regenerative medicine.

Despite the promising results of existing studies, several knowledge gaps remain in the field of EV-mediated wound healing. While the role of MSC-EVs in promoting tissue repair is well documented, the specific mechanisms underlying their effects are not yet fully understood. Further research is needed to elucidate the precise molecular pathways activated by EVs and their interactions with other signaling cascades during the healing process [20,21]. Meanwhile, in Malaysia, difficult-to-heal wounds, such as chronic ulcers and severe burns, are a significant health concern, particularly for individuals with diabetes, vascular diseases, and infections. EV therapies in Malaysia are still in the early stages of research and development. Further research is necessary before they can be approved.

This study aimed to isolate and characterize umbilical cord MSC-derived EVs (UC-MSC-EVs) using tangential flow filtration (TFF). The effects of these EVs on DF proliferation, mitochondrial activity, migration, and ECM production were assessed, with a focus on key components such as collagen and fibronectin, which are critical for wound healing. Additionally, the immunomodulatory effects of EVs in reducing inflammatory cytokine secretion from peripheral blood mononuclear cells (PBMCs) were explored. These findings aim to establish UC-MSC-EVs as a promising therapeutic tool for enhancing cutaneous wound healing, with future in vivo studies planned to validate their clinical potential.

## 2. Materials and Methods

### 2.1. Ethics

This research was conducted with ethics approval from the Research Ethics Committee of the National University of Malaysia (RECUKM; approval code: JEP-2023-279).

### 2.2. Cell Culture

Dermal fibroblasts (DFs) were chosen for the wound healing study because they play a key role in the wound healing process. Fibroblasts are the most abundant cells in the dermis, and are responsible for producing ECM components like collagen, which are essential for wound repair and remodeling. DFs were seeded at a density of 5000 cells per well to create a cell culture environment that closely mimicked the physiological conditions relevant to wound healing. This specific cell density is commonly used in wound healing research, because it facilitates adequate interaction between the cells and EVs, while preventing overcrowding that could alter cell behavior, both of which are essential for assessing the potential of UC-MSC-EVs in enhancing wound healing. DFs were obtained from the Department of Tissue Engineering and Regenerative Medicine (DTERM) primary cell bank (ethics approval code: FF-2015-376). Briefly, the revived DFs were cultured in Dulbecco’s Modified Eagle’s Medium (DMEM) and Ham’s F-12 nutrient mixture medium (Sigma-Aldrich, St. Louis, MO, USA) supplemented with 10% human platelet lysate (HPL), prepared in-house using the method described previously [22,23]. The cells were maintained at 37 °C in a 5% CO_2_ atmosphere, with the medium changed every 2–3 days.

UC-MSCs, previously characterized as being over 95% positive for CD73, CD90, and CD105, and less than 2% positive for CD34, CD45, CD11b, CD14, CD19, and HLA-DR, were obtained from the DTERM primary cell bank. The cells were grown in low-glucose DMEM at a 10% concentration of HPL (Sigma-Aldrich, St. Louis, MO, USA). The medium was further supplemented with 1% antibiotic–antimycotic solution (Gibco, New York, NY, USA) to prevent microbial contamination. The cells were reactivated in this medium and incubated at 37 °C in a humidified atmosphere containing 5% CO_2_. The culture medium was replaced every 2–3 days. To collect the conditioned medium, the cells were incubated in a serum-free medium for 48 h. The conditioned medium was then collected and filtered using a sterile 0.22 μm filter. To preserve its integrity, the filtered medium was stored at −80 °C until required for further research or testing.

### 2.3. EV Isolation and Characterization

The collected conditioned medium was centrifuged to remove detached cells, large debris, and large particle contaminants, and concentrated using Minimate Tangential Flow Filtration (TFF) Capsules with Omega Membranes-300K (OA300C12, Pall Corporation, New York, NY, USA) and a Minimate Tangential Flow Filtration System (Pall Corporation, New York, NY, USA). The pump speed was adjusted to maintain a feed pressure of 30 psi, a retentate pressure of 25 psi, a filtrate pressure of 0 psi, and a transmembrane pressure (TMP) of 2.5 psi. The conditioned medium was concentrated to a final volume of 10 mL. This was followed by buffer recirculation and diafiltration with PBS to dislodge impurities and EVs lodged in the filter membrane; this was repeated three times to yield a final 10 mL volume. The concentrated EVs were sterile filtered using a 0.22 μm filter, and stored at −80 °C for subsequent analysis and experiments. Minimate Tangential Flow Filtration Capsules were cleaned for reuse by pre-washing with sterile PBS (pH 7.4), followed by 20% ethanol (Sigma-Aldrich, St. Louis, MO, USA), and rinsing with sterile PBS. Post-processing, the filters were washed with sterile PBS and 0.1 M NaOH (Sigma-Aldrich, St. Louis, MO, USA), then stored at 4 °C, ensuring the integrity and purity of the conditioned medium for subsequent experiments. The isolated EVs were characterized using a bicinchoninic acid assay (Thermo Fisher Scientific, Waltham, MA, USA), nanoparticle tracking analysis (NTA; NanoSight NS300, Malvern Panalytical, Malvern, UK), and a high-resolution transmission electron microscope (HRTEM; JEOL, Tokyo, Japan), confirming the identity and purity of the EVs.

### 2.4. Cell Morphology and Viability

The morphological features of DFs treated with 25, 50, 75, and 100 µg/mL of EVs were examined every three days using an inverted light microscope. Cultured DFs were grown to 80–90% confluence, before being detached with 0.05% trypsin-EDTA (Sigma-Aldrich, St. Louis, MO, USA), and the cell number and viability were quantified using a hemocytometer and trypan blue. Viability was determined using the following formula:

Cell viability (%) = (Total live cells/Total live + dead cells) × 100%

### 2.5. Cell Proliferation Assay via CCK8

The proliferation rate of DFs upon exposure to various concentration of EVs (25, 50, 75 and 100 µg/mL) was determined using a CCK-8 assay (Elabscience, Wuhan, China). DFs were seeded at a density of 5000 cells/well in 96-well plates (BD Bioscience, Franklin Lakes, NJ, USA), and treated with EVs or a vehicle control (PBS). At 24 and 48 h, cell metabolic activity was measured using the CCK-8 assay, following the manufacturer’s instructions. The average optical density (OD) values were calculated, and the cell proliferation rate was determined using the following formula:

Cell proliferation rate (%) = (OD of EV treated cells/OD of untreated cells) × 100%

The selected time points for assessing cell proliferation, migration, and ECM production were 24 and 48 h, based on established wound healing kinetics. These time points are crucial for the early stages of wound healing, during which key cellular responses such as metabolic activity, proliferation, and ECM remodeling are most pronounced. The 24 h mark allows for an initial evaluation of the effects of EVs on these cellular responses. In contrast, the 48 h mark offers insights into the sustained effects of EVs and their capacity to influence long-term cellular functions, including migration and ECM synthesis.

### 2.6. Cell Viability Assay via MTT

The cell viability rate of DFs upon exposure to various concentration of EVs (25, 50, 75 and 100 µg/mL) was determined using an MTT assay (Sigma-Aldrich, St. Louis, MO, USA). DFs were seeded at a density of 5000 cells/well in 96-well plates, and treated with EVs or a vehicle control (PBS). At 24 and 48 h, mitochondrial function was assessed using the MTT assay, which measures the activity of mitochondrial NAD(P)H-dependent oxidoreductases. These enzymes reduce MTT to formazan, a colored product whose absorbance is directly proportional to the mitochondrial metabolic activity of viable cells. The assay was performed following the manufacturer’s instructions, and the formazan product was quantified by measuring absorbance at 570 nm using a microplate reader. The results reflect the mitochondrial functionality and viability of the treated cells. The average OD values were calculated, and the cell survival rate was determined using the following formula:

Cell viability rate (%) = (OD of EV treated cells/OD of untreated cells) × 100%

### 2.7. Cell Migration Assay

A scratch wound assay was performed to assess the migration of DFs. A monolayer of cells was cultured to full confluence before initiating the wound assay, ensuring proper experimental conditions for accurate cell migration analysis. The cells were scratched with a sterile pipette tip to create a linear wound approximately 2 mm in diameter, and the culture was washed to remove any debris. Following this, the cells were treated with EVs at concentrations of 25, 50, 75, and 100 µg/mL. Wound closure was monitored by live imaging, with images captured every hour, for 72 h, at three distinct spots along the wound edge, using a Nikon A1R-A1 confocal laser scanning microscope (Tokyo, Japan). The captured images were then analyzed using NIS Elements AR3.1 software (Nikon, Tokyo, Japan), which allowed for quantification of the wound gap and assessment of the migration rate over time. The percentage of wound closure was determined using the following formula:

$$Wound\;closure\;rate\;(\%)=\frac{\left(Wound\;area\;at\;time\;0)-(Wound\;area\;at\;time\;x\right)}{Wound\;area\;at\;time\;0}\times 100\%$$

where “time 0” represents the initial wound area right after the scratch is made, and “time x” represents the wound area at a specific time point during the experiment. This was followed by normalization of the wound closure percentage of each treated group to the untreated control group at the same time point, in order to calculate the fold change, using the following formula:

$$Fold\;change=\frac{Wound\;Closure\;Percentage\;of\;Treated\;Group\;at\;Time\;x}{Wound\;Closure\;Percentage\;of\;Control\;Group\;at\;Time\;x}$$

### 2.8. Reactive Oxygen Species (ROS) Assay

DFs were seeded at a density of 5000 cells per well in 96-well plates and treated with EVs at concentrations of 25, 50, 75, and 100 µg/mL, or a vehicle control (PBS). ROS generation was assessed at 24 and 48 h using the DCFDA/H2DCFDA Cellular ROS Assay Kit (Abcam, Waltham, MA, USA), following the manufacturer’s instructions. This assay uses the fluorescent probe DCFDA, which diffuses into the cells and is deacetylated by intracellular esterases to form H2DCFDA. The probe reacts with ROS to produce a fluorescent signal, which correlates with intracellular ROS levels. Fluorescence intensity, indicating oxidative stress, was measured using a fluorescence microplate reader at excitation/emission wavelengths of 485/535 nm.

### 2.9. Cytokine Assay

Peripheral blood mononuclear cells (PBMCs) were collected from healthy donor blood via Ficoll-Paque density gradient centrifugation (GE Healthcare, Chicago, IL, USA), and washed twice with PBS. The PBMCs were cryopreserved in liquid nitrogen, and thawed at 37 °C for use in experiments. After thawing, the cells were suspended in RPMI medium (Sigma-Aldrich, St. Louis, MO, USA) and centrifuged to remove dimethylsulfoxide (DMSO; Sigma-Aldrich, St. Louis, MO, USA), then resuspended in RPMI medium supplemented with 10% FBS (Sigma-Aldrich, St. Louis, MO, USA), at a density of approximately 5 million cells/mL. For the immunomodulatory effect assessment, 300,000 cells in 200 µL were seeded into 96-well plates and incubated at 37 °C in a 5% CO_2_ atmosphere. The PBMCs were stimulated with lipopolysaccharide (LPS; 1 µg/mL; Sigma-Aldrich, St. Louis, MO, USA) for 24 h, with or without UC-MSC-EVs, at concentrations of 25, 50, 75, and 100 µg/mL. After 48 h, the conditioned medium was collected and stored at −20 °C for subsequent cytokine quantification using ELISA. The cytokines measured included IL-6, IL-33, TNF-α, and TGF-β, using the LEGEND MAX™ Human IL-6 ELISA Kit (BioLegend, San Diego, CA, USA), LEGEND MAX™ Human IL-33 ELISA Kit (BioLegend, San Diego, CA, USA), LEGEND MAX™ Human TNF-α ELISA Kit (BioLegend, San Diego, CA, USA), and LEGEND MAX™ Total TGF-β1 ELISA Kit (BioLegend, San Diego, CA, USA). The sensitivity of these assays was 0.23 ng/mL for IL-6 (standard range: 7.8–500 pg/mL), 4.14 pg/mL for IL-33 (standard range: 15.6–1000 pg/mL), 0.8 pg/mL for TNF-α (standard range: 15.6–1000 pg/mL), and 0.23 ng/mL for TGF-β1 (standard range: 1.56–100 ng/mL). The assays were performed following the manufacturer’s protocols, which included coating plates with capture antibodies, blocking, adding samples or standards, and using a secondary antibody conjugated to an enzyme for detection. Substrate addition resulted in a color change, and the absorbance was measured to determine cytokine concentrations.

### 2.10. Growth Factor Assay

The concentrations of growth factors, specifically BDNF and VEGF, in the medium from DFs incubated with various concentrations of UC-MSC-EVs (25, 50, 75, and 100 µg/mL) were measured using BioLegend ELISA kits (BioLegend, San Diego, CA, USA). For BDNF, the sensitivity was 27.5 pg/mL, with a standard range of 156.3–1000 pg/mL, and for VEGF, the sensitivity was 4.1 ± 2.2 pg/mL, with a standard range of 23.4–1500 pg/mL. The medium samples were diluted with the provided diluent and added to antibody-coated wells, followed by a 2 h incubation. Detection antibodies and HRP-avidin were added sequentially, followed by incubation for 1 h and 30 min, respectively. Afterward, TMB substrate solution was added to each well, followed by incubation for 10 min at room temperature in the dark. The reaction was stopped with a stop solution, and absorbance was measured at 450 nm using a spectrophotometric multi-well plate reader.

Meanwhile, IGF and KGF levels were measured using ELK Biotechnology ELISA kits (ELK Biotechnology, Wuhan, China). For IGF, the sensitivity was 0.067 ng/mL, with a detection range of 0.16–10 ng/mL, and for KGF, the sensitivity was 6.1 pg/mL, with a detection range of 15.63–1000 pg/mL. To measure these factors, 100 μL of standard working solution or sample was added to the plate, followed by incubation at 37 °C for 80 min. After washing, biotinylated antibody solution was added and the plate was incubated for 50 min, followed by incubation with streptavidin-HRP solution for another 50 min. TMB substrate solution was added and the plate was incubated at 37 °C for 20 min; then, the reaction was stopped with stop reagent. Absorbance was immediately measured at 450 nm using a spectrophotometric multi-well plate reader, ensuring that no bubbles or contaminants were present on the plate.

### 2.11. Quantitative Gene Expression Analysis by Real-Time PCR

Real-time PCR was used to analyze the expression of type I collagen, type III collagen, fibronectin, and glyceraldehyde 3-phosphate dehydrogenase (GAPDH) in the DFs treated with various concentrations of UC-MSC-EVs (25, 50, 75 and 100 µg/mL). Primers were designed using Primer 3 software and the GeneBank database (Table 1). Real-time PCR reactions were performed with 100 ng of total RNA, 400 nM of each primer, and the iScript One-Step RT-PCR kit with SYBR Green (Bio-Rad, Hercules, CA, USA), based on the manufacturer’s instructions. Reactions were run on a Bio-Rad iCycler with the following profile: cDNA synthesis at 50 °C for 30 min; pre-denaturation at 94 °C for 2 min; and PCR amplification for 38 cycles (30 s at 94 °C, 30 s at 60 °C, and 30 s at 72 °C). A melt curve analysis followed to confirm reaction specificity. Gene expression was normalized to GAPDH.

### 2.12. Statistical Analysis

Statistical analyses were conducted using GraphPad Prism version 10 (GraphPad Software, La Jolla, CA, USA). One-way ANOVA was used to evaluate differences among the treatment groups. For pairwise comparisons, Tukey’s Honest Significant Difference (HSD) post hoc test was applied when appropriate. Statistical significance was defined as *p* < 0.05.

## 3. Results

### 3.1. Characterization of UC-MSC-EVs

**Figure 1:**
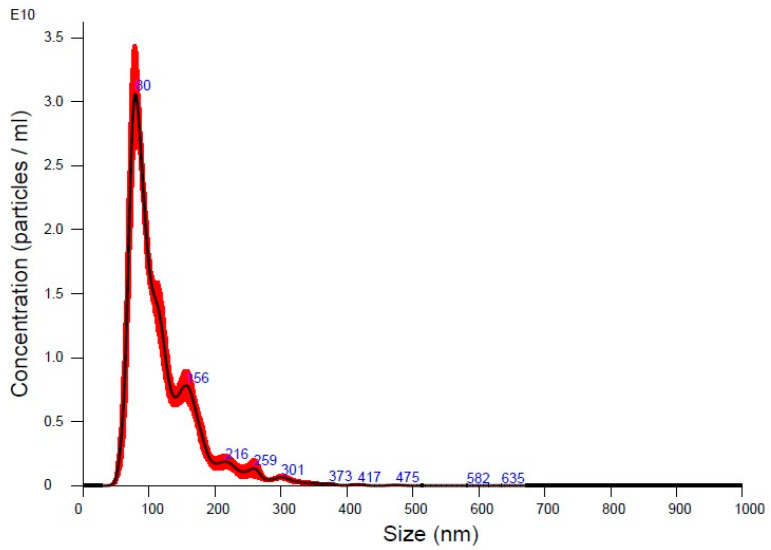
The particle size distribution graph shows the concentration of EVs measured through NTA. The dominant EV population peak is at approximately 80 nm (highest peak), indicating that this is the most abundant size in the sample. There are smaller peaks at larger sizes, such as around 216 nm and beyond, suggesting a heterogeneous population of vesicles, though these larger particles are less concentrated.

**Figure 2:**
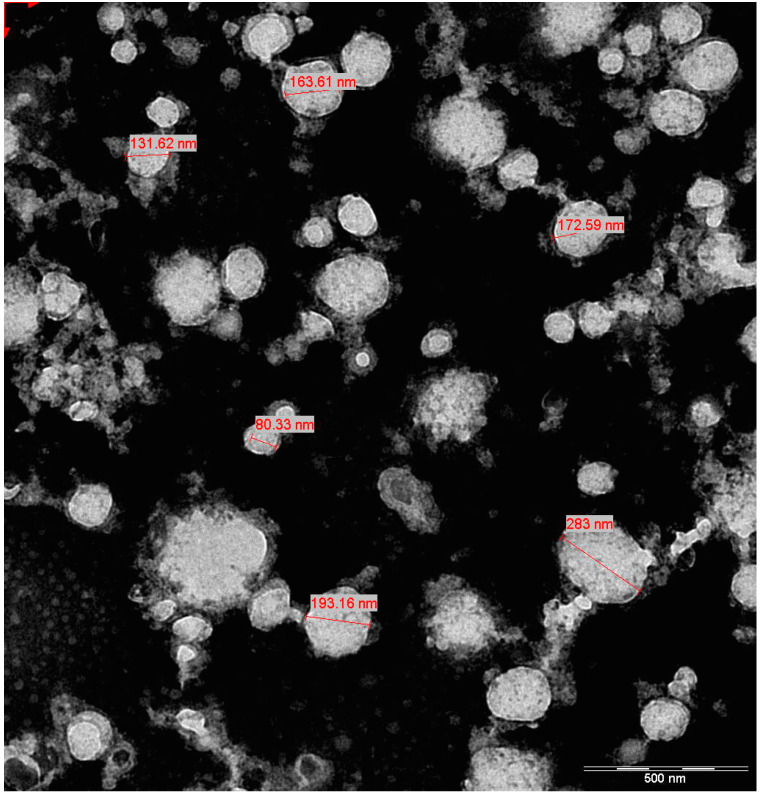
TEM visualization of EVs. The annotated size measurements of various vesicles, ranging from 80 nm to 283 nm, match the expected size range of EVs, indicating that the image provides accurate dimensional information. With a scale bar indicating 500 nm, the image effectively illustrates the heterogeneity in vesicle sizes, providing valuable insights into the characteristics of the EV population under investigation.

The NTA of UC-MSC-EVs showed a mean particle size of 119.2 nm, with a mode size of 81.5 nm, indicating a population primarily within the exosome size range (Figure 1). The size distribution, with D10, D50, and D90 values of 72.5 nm, 101.1 nm, and 184.8 nm, respectively, reflects a typical EV population (Table 2). The concentration of 1.83 × 10^12^ particles/mL suggests successful EV isolation with a high particle yield. Overall, the data confirmed the presence of a well-characterized EV sample that was suitable for further experimentation. Meanwhile, TEM analysis showcased typical EV morphology and a size range from approximately 80 nm to 283 nm, aligning with the expected dimensions for exosomes and microvesicles (Figure 2). The smallest vesicles, measuring around 80.33 nm, are consistent with exosomal sizes, while the largest, at approximately 283 nm, likely represent microvesicles or larger EVs. These findings illustrate the heterogeneous nature of the EV population.

### 3.2. Morphological Changes in DFs After Exposure to EVs

**Figure 3:**
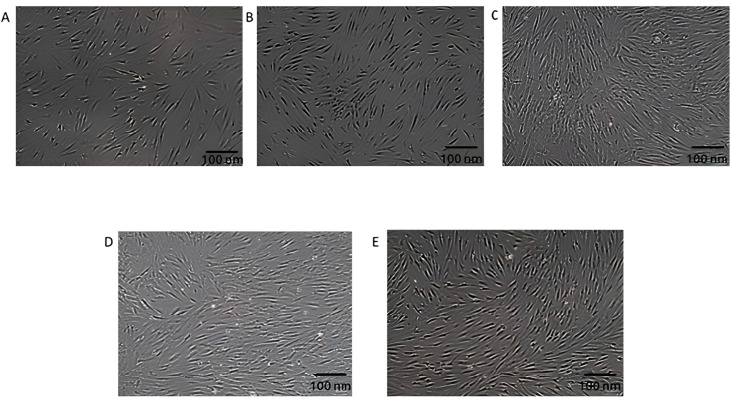
Structural changes in DFs after exposure to varying concentrations of EVs. (**A**) Untreated control cells display a typical elongated, spindle-shaped morphology. (**B**) DFs exposed to 25 µg/mL of EVs show a slight increase in cell density, while maintaining a similar morphology. (**C**) DFs treated with 50 µg/mL of EVs exhibit the most pronounced effects, characterized by denser and healthier cell populations, suggesting enhanced proliferation; also, the space between each cell is reduced compared to (**A**,**B**). (**D**,**E**) DFs exposed to 75 µg/mL (**D**) and 100 µg/mL (**E**) of EVs retain fibroblast-like morphology, but do not show further improvement in morphology or density compared to DFs exposed to 50 µg/mL of EVs. All images were captured at a resolution of 100 nm.

Figure 3 demonstrates DFs exposed to varying concentrations of EVs. Figure 3A shows untreated cells with typical elongated, spindle-shaped morphology and a well-spread appearance. Figure 3B depicts DFs exposed to 25 µg/mL of EVs, showing a slight increase in cell density, though the overall morphology remains similar to that of the untreated cells. Figure 3C, representing cells treated with 50 µg/mL of EVs, shows the most significant impact, with DFs appearing denser and healthier, indicating enhanced proliferation or activity. Figure 3D,E show cells exposed to higher EV concentrations (75 and 100 µg/mL), where the morphology is still fibroblast-like, but with no further improvement compared to Figure 3C. The optimal effects on fibroblast morphology and density are observed in Figure 3C, indicating that this concentration of EVs yields the best results. Table 3 provides the observations of EV-treated DFs based on morphological changes. Meanwhile, at 24 h, for untreated control cells with 100% viability, there are 150,000 live cells, which equates to a total cell population (live + dead) of 150,000. For 25 µg/mL at 24 h (120% viability), there are 180,000 live cells, and the total cell count remains at 150,000. At 50 µg/mL (140% viability), the number of live cells increases to 210,000, and the total cell count is still 150,000. For 75 µg/mL (100% viability), the number of live cells equals 150,000, matching the total cell population. At 100 µg/mL (90% viability), the number of live cells is 135,000, and the total number of cells remains at 150,000. For 48 h, at 25 µg/mL (125% viability), the number of live cells increases to 187,500, with a total cell count of 150,000, while at 50 µg/mL (145% viability), the number of live cells is 217,500, with the same total cell count. At 75 µg/mL (100% viability), the number of live cells is 150,000, and the total cell count remains unchanged, at 150,000; and for 100 µg/mL (90% viability), the number of live cells is 135,000, with a total cell count of 150,000. These findings highlight that 50 µg/mL of EVs induced the most pronounced cell proliferation at both 24 and 48 h, while higher concentrations (75 and 100 µg/mL) did not enhance viability, potentially indicating a saturation effect or cytotoxicity at higher doses.

### 3.3. EVs Increase Proliferation of DFs

**Figure 4:**
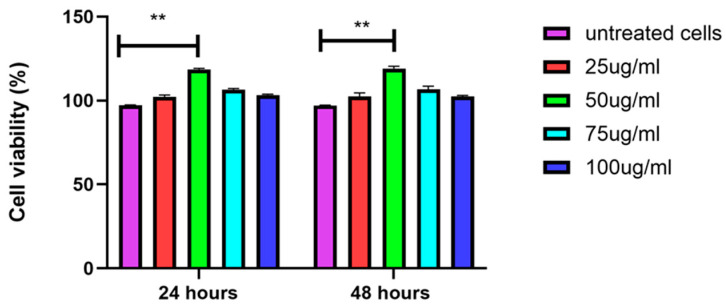
Increase in metabolic activity of EV-treated DFs. Concentration of 50 µg/mL shows good metabolic activity, indicating higher proliferation of DFs compared to other concentrations of EVs. Data are mean ± S.E.M. of *n* = 3 independent experiments carried out in triplicates. ** indicates *p* < 0.01.

The CCK-8 assay measures the metabolic activity of cells, which is an indicator of cell viability and proliferation. The results show that treatment with a concentration 50 µg/mL significantly enhances the metabolic rate of the cells at both 24 and 48 h compared to the untreated control. At other concentrations (25, 75 and 100 µg/mL), the metabolic rate is less increased, but remains higher than in the untreated cells. The significant differences (*p* < 0.01) suggest a concentration-dependent effect, with optimal metabolic enhancement at 50 µg/mL (Figure 4).

### 3.4. EVs Increase Viability of DFs

**Figure 5:**
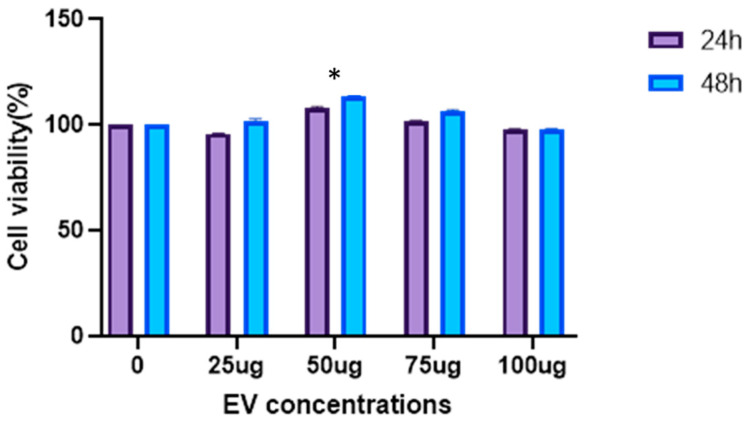
Increase in cell mitochondrial activity via MTT assay after exposure with various EV concentrations. Treatment with 50 µg/mL of EVs significantly increased mitochondrial activity at both time points, with other concentrations (25, 75 and 100 µg/mL) also increasing mitochondrial activity, but to a lesser extent. Data are mean ± S.E.M. of *n* = 3 independent experiments carried out in triplicates. * indicates *p* < 0.05.

The MTT assay results, which measure mitochondrial activity as an indicator of cell viability, show that treatment at the concentration of 50 µg/mL significantly increases mitochondrial activity compared to untreated cells at both 24 and 48 h. The untreated control cells are set at 100% viability. At 24 h, cells treated with 50 µg/mL show the highest increase in mitochondrial activity, followed by the 25 µg/mL, 75 µg/mL, and 100 µg/mL treatments, all showing higher activity compared to untreated cells. At 48 h, a similar trend is observed, with 50 µg/mL-treated cells showing the highest mitochondrial activity, followed by cells treated with 25 µg/mL, 75 µg/mL, and 100 µg/mL of EVs. Figure 5 provides the treatment enhances mitochondrial activity in a concentration-dependent manner, with the greatest effect observed at 50 µg/mL.

### 3.5. EVs Induce Migration of DFs

**Figure 6:**
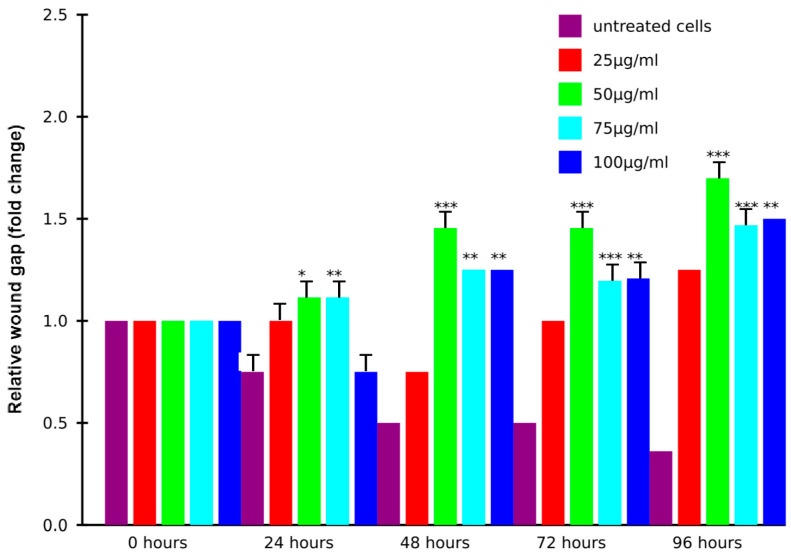
Induction of cell migration in concentration-dependent manner by EVs. Migration assay showing fold change in wound gap closure over time (0, 24, 48, 72, and 96 h) under different treatment conditions. Untreated cells exhibit negligible migration, while treatments with 25 µg/mL, 50 µg/mL, 75 µg/mL, and 100 µg/mL demonstrate progressively enhanced cell migration. Treated groups exhibit increased wound gap measurements, particularly at 50 μg/mL, which shows a 2.2-fold increase compared to the control. Higher concentrations (75 and 100 μg/mL) also caused significant increases, though less pronounced than 50 μg/mL. Data are mean ± S.E.M. of n = 3 independent experiments carried out in triplicates. * indicates *p* < 0.05, ** indicates *p* < 0.01, and *** indicates *p* < 0.001 compared to the untreated control.

**Figure 7:**
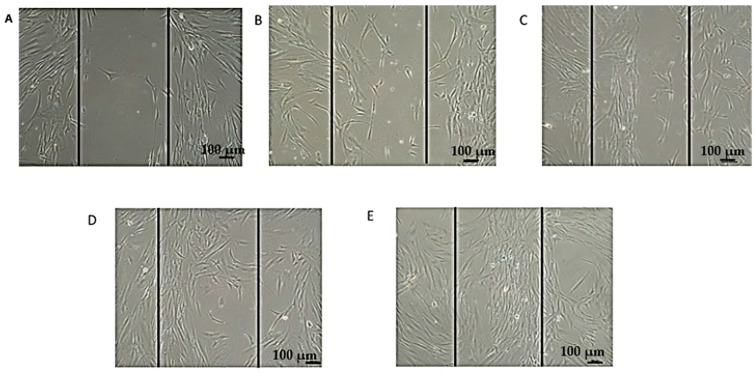
Representative images of cell migration during wound healing assay after treatment with 50 µg/mL of EVs. (**A**) 0 h: initial wound gap immediately after scratch is created. (**B**) 24 h: cells begin migrating into wound area. (**C**) 48 h: increased cell migration is observed, resulting in partial closure of wound gap. (**D**) 72 h: substantial closure of wound gap occurs due to enhanced cell migration and proliferation. (**E**) 96 h: wound gap is nearly completely closed, demonstrating effective cell migration and repair. Scale bar: 100 µm.

The cell migration assay shows the progression of the wound gap (normalized fold change) over time (0, 24, 48, 72, and 96 h) across different treatment conditions. Untreated cells show minimal wound closure, suggesting limited cell migration. This time-course experiment over 96 h demonstrates that the treatment significantly increases the wound gap in a time-dependent manner, with the most pronounced effects observed at 96 h. While untreated control cells maintain the lowest wound gap throughout, treated groups exhibit increased wound gap measurements, particularly at 50 μg/mL, which shows a 2.2-fold increase compared to the control. Higher concentrations (75 and 100 μg/mL) also caused significant increases, though less pronounced than 50 μg/mL (Figure 6). In addition, the wound healing assay shows the progression of cell migration over 0, 24, 48, 72, and 96 h. At 0 h (A), the wound gap is clearly visible, with no significant cell migration. By 24 h (B), cells begin to migrate into the gap, partially reducing its size. At 48 h (C), more substantial coverage is observed, with cells aligning and moving toward the wound center. By 72 h (D), the gap is significantly reduced, indicating accelerated migration and proliferation. At 96 h (E), the wound is nearly closed, with dense cellular coverage and minimal visible gap, demonstrating progressive and effective wound closure over time (Figure 7).

### 3.6. Higher Concentrations of EVs Induce Secretion of ROS

**Figure 8:**
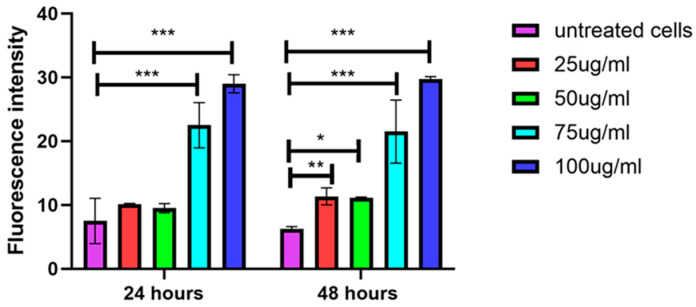
Dose-dependent increase in ROS production with higher EV concentrations. ROS assay results show that ROS production in treated cells increases with higher doses of EVs, as indicated by elevated fluorescence intensity. Untreated cells exhibit lowest ROS levels, while higher EV concentrations significantly increase ROS generation. Data are mean ± S.E.M. of *n* = 3 independent experiments carried out in triplicates. * indicates *p* < 0.05, ** indicates *p* < 0.01, *** indicates *p* < 0.001 compared to the untreated control.

The ROS assay results demonstrate a dose-dependent increase in ROS production in treated cells, as indicated by rising fluorescence intensity. At both 24 and 48 h, untreated cells exhibit the lowest ROS levels, while treatment with higher concentrations (75 µg/mL and 100 µg/mL) significantly elevates ROS levels, with 100 µg/mL producing the highest fluorescence. Lower concentrations (25 µg/mL and 50 µg/mL) result in moderate ROS generation; however, both concentrations show significant differences at 48 h compared to the untreated cells. Statistically significant differences (*p* < 0.001) between treated and untreated groups suggest that EVs induce oxidative stress, particularly at higher doses (Figure 8).

### 3.7. Secretion of Inflammatory Cytokines in DFs upon Exposure of EVs

**Figure 9:**
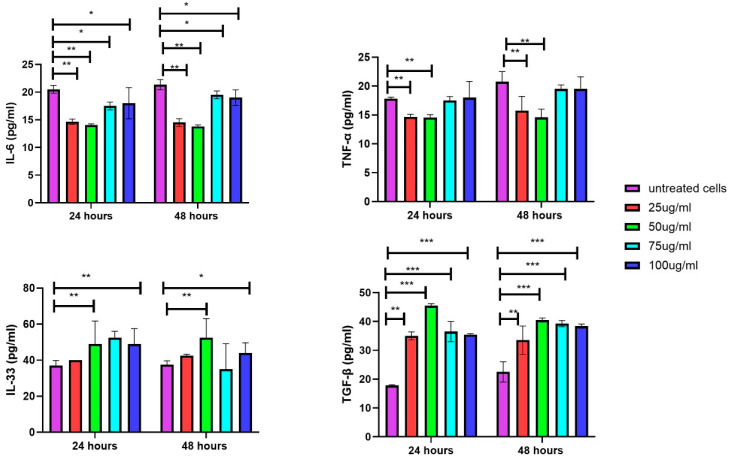
Increase in inflammatory cytokines in cells treated with varying EV concentrations for 24 and 48 h. DFs treated with 25 and 50 μg/mL of EVs demonstrated more pronounced decrease in IL-6 and TNF-α secretions compared those treated with higher concentrations (75 and 100 μg/mL). As for IL-33 and TGF-β, higher increments in levels were detected in DFs treated with 50, 75, and 100 μg/mL of EVs compared to those treated with 25 μg/mL of EVs. Data are mean ± S.E.M. of *n* = 3 independent experiments carried out in triplicates. Statistically significant differences are labeled as * indicating *p* < 0.05, ** indicating *p* < 0.01, and *** indicating *p* < 0.001 compared to the untreated control.

The inflammatory assays reveal cytokine secretion levels, including IL-6 and TNF-α, in cells exposed to different treatment conditions at 24 and 48 h. At both time points, untreated cells exhibit the highest levels of IL-6 and TNF-α. Treatment with 25 µg/mL and 50 µg/mL significantly reduces IL-6 and TNF-α production, while higher concentrations (75 µg/mL and 100 µg/mL) show a less pronounced reduction, with levels approaching those of untreated cells. This suggests that lower EV concentrations are more effective at suppressing IL-6 and TNF-α, whereas higher EV concentrations have a reduced impact on these cytokine suppressions over time. The IL-33 and TGF-β assay results show increased secretion in DFs treated with 50, 75, and 100 µg/mL of EVs at both time points compared to the untreated cells. Although treatment with lowest EV concentration tested, i.e., 25 µg/mL, also raises the secretion of these cytokines, the effect is less pronounced (Figure 9).

### 3.8. Increase in Growth Factors in DFs upon Exposure to EVs

**Figure 10:**
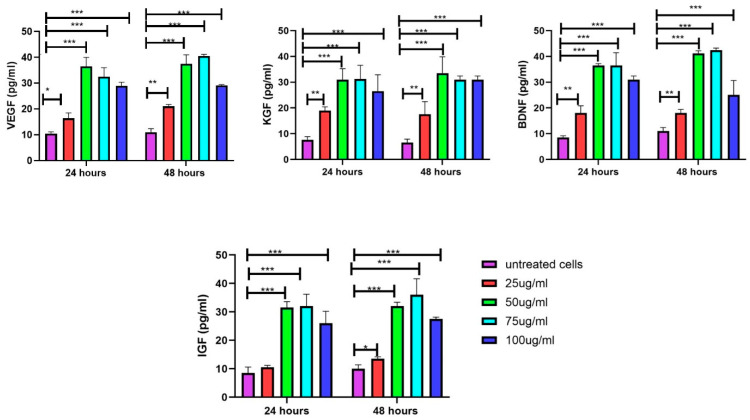
Treatment concentration of EVs and duration significantly influenced VEGF, BDNF, KGF, and IGF levels. EV treatment increased growth factor secretions, with 50 and 75 μg/mL treatments consistently showing highest levels at both time points, followed by 100 μg/mL treatment, with 25 μg/mL treatment demonstrating lowest levels. Data are mean ± S.E.M. of *n* = 3 independent experiments carried out in triplicates. Statistically significant differences are labeled as * indicating *p* < 0.05, ** indicating *p* < 0.01, and *** indicating *p* < 0.001 compared to the untreated control.

The VEGF levels were significantly influenced by both treatment concentration and duration. At 24 h, all treatment concentrations (25–100 μg/mL) led to a substantial increase in VEGF levels compared to untreated cells (*p* < 0.001). The 50 and 75 μg/mL treatments resulted in the highest VEGF levels, approximately four times greater than in untreated cells. By 48 h, this effect became even more pronounced, with the 50, 75, and 100 μg/mL treatments maintaining significantly elevated VEGF levels (*p* < 0.001) relative to untreated cells. Additionally, the 25 μg/mL treatment showed a notable increase (*p* < 0.01) at 48 h. Among all concentrations, the 50 and 75 μg/mL treatments consistently produced the highest VEGF levels at both time points, highlighting their potential effectiveness. Similar trends were observed for BDNF, KGF, and IGF where treatment concentration and duration significantly influenced their levels. Overall, the 50 and 75 μg/mL concentrations consistently emerged as the most effective for enhancing the levels of growth factors at both time points (Figure 10).

### 3.9. Increase in Gene Expression of Extracellular Matrix Proteins upon Exposure to EVs

**Figure 11:**
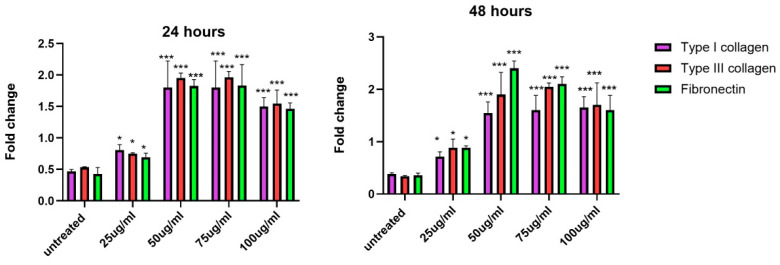
Increase in gene expression levels of type I collagen, type III collagen, and fibronectin in response to varying concentrations of EVs. At 24 and 48 h, mild increases in ECM gene expression are observed at 25 µg/mL, with highest fold change seen at 50 and 75 µg/mL. Data are mean ± S.E.M. of *n* = 3 independent experiments carried out in triplicate. Data were compared with untreated cells. Statistically significant differences are labeled as * indicating *p* < 0.05, and *** indicating *p* < 0.001 compared to the untreated control.

The fold changes in the gene expression of three key ECMs, including type I collagen, type III collagen, and fibronectin, were evaluated across different concentrations of EVs at 24 and 48 h. At both 24 and 48 h, the lowest concentration of 25 µg/mL showed mild increases in the expressions of all three ECM proteins compared to the untreated control. Starting from 50 µg/mL, there was a noticeable increase in the expression of all proteins. At 75 µg/mL and 100 µg/mL, the expression of type I collagen, type III collagen, and fibronectin significantly increased. Overall, the results indicate that EVs stimulate the gene expression of ECM proteins in a time- and concentration-dependent manner. The most notable effects were observed at 50–75 µg/mL concentrations, particularly at the 48 h mark, when the highest fold changes in gene expression were recorded for all three proteins. This trend is consistent with the prolonged incubation of EVs, which appears to amplify the stimulatory effects on ECM protein production (Figure 11).

## 4. Discussion

Recent advances in the field of regenerative medicine have underscored the significance of UC-MSC-EVs in influencing DF function. The findings aim to elucidate the mechanisms by which UC-MSC-EVs affect cellular metabolism, mitochondrial activity, migration, oxidative stress, inflammatory cytokine secretion, growth factor production, and ECM synthesis. Through these mechanisms, EVs display their therapeutic potential in tissue regeneration and wound healing, offering new pathways for innovative treatment strategies [3].

The characterization of EVs with a mean size of 118.6 nm confirmed their exosomal nature, consistent with their function as mediators of intercellular communication [24,25,26]. The observed size heterogeneity in EVs suggests the presence of both exosomes and microvesicles within the sample, with implications for their functional versatility [27]. Exosomes, due to their smaller size, are more likely to facilitate targeted delivery of biomolecules, making them a valuable tool for therapeutic applications, particularly in wound healing and tissue regeneration [28]. On the other hand, microvesicles, along with apoptotic bodies, tend to be larger, and may be involved in more complex mechanisms, such as immune responses, apoptosis, and direct cellular repair [29,30]. The presence of both exosomes and microvesicles could enhance the therapeutic potential of the EVs, as they may collectively influence multiple cellular pathways simultaneously [31].

The concentration-dependent increase in metabolic and mitochondrial activity, especially at lower concentrations of EVs (25–50 µg/mL), suggests that these EVs have the potential to substantially enhance the energy functions of DFs. This improvement in cellular energetics is indicative of the role of EVs in stimulating metabolic processes and mitochondrial efficiency, contributing to improved cell function and potentially aiding in wound healing and tissue regeneration. Previous studies have suggested that EVs may deliver mitochondrial components, proteins, or metabolic enzymes to recipient cells, which could underlie this observed enhancement of energy production [32]. However, the reduced effects observed at higher concentrations (75–100 μg/mL) are likely influenced by several factors, including EV concentration, source, and delivery method [33]. While EVs have demonstrated significant potential in promoting wound healing, higher concentrations do not always enhance their effects, and may even be detrimental in some cases [34]. Research suggests that EVs exhibit diminishing returns in wound healing, where low to moderate concentrations enhance healing, but excessive doses can impair responses. Garima et al. (2023) reported that higher EV concentrations might provoke inflammation or disrupt cellular responses [35]. Similarly, Sarcinella et al. (2023) highlighted that elevated EV levels could disturb the balance between pro-inflammatory and anti-inflammatory signals, potentially leading to adverse outcomes. These findings emphasize the need for optimizing EV concentrations to achieve effective therapeutic outcomes in wound healing [36].

The wound healing assay results provide critical insights into the dynamic process of cell migration and proliferation in response to a wound. The time-dependent progression of DF wound closure observed highlights key phases of the wound healing mechanism [33]. Increased levels of EVs deliver a higher concentration of signaling molecules to target cells, which can enhance cell migration by activating pathways such as EMT. However, at higher concentrations, the elevated EV levels may also induce ROS production, triggering cellular stress responses. This oxidative stress can impair mitochondrial function, leading to a decrease in metabolic activity [36]. EVs from MSCs have been reported to enhance skin cell migration and expedite proliferation to the wound site [20]. These findings underscore the potential of EVs as therapeutic agents for wound healing, with the optimization of delivery systems key to maximizing their efficacy, especially in conditions like diabetic wound healing [34].

An intriguing finding is the dose-dependent increase in ROS levels, particularly at higher concentrations of EVs, which can lead to varying effects on cell bioactivities. While high ROS levels are typically associated with cellular stress, moderate increases can serve as signals for adaptive responses that promote cell survival and regeneration, a phenomenon known as hormesis [37]. High concentrations of exosomes can induce elevated ROS levels, potentially due to their cargo, which includes molecules that trigger oxidative stress pathways. Although excessive ROS can impair endothelial cell function [38], lower levels can promote cell proliferation, while very high levels can lead to cellular dysfunction and death. In the wound healing process, excessive ROS can reduce the number of fibroblasts, keratinocytes, and endothelial cells [39]. Interestingly, MSC-derived EVs have a protective role, as they not only reduce ROS accumulation, but also enhance antioxidant signaling pathways, thereby mitigating oxidative damage. Exosomes also help to maintain cellular homeostasis by removing harmful cytoplasmic DNA, preventing its accumulation and triggering ROS-dependent DNA damage and cellular senescence [37,38]. This highlights the dual role of ROS: acting as a promoter of cell survival in moderate amounts, and as a trigger for oxidative damage at high levels. Thus, further research is needed in order to understand the conditions under which EV-induced ROS enhance or inhibit cell proliferation across different cell types [40].

The critical balance between inflammation and regeneration during wound healing involves a well-coordinated response that ensures effective healing while preventing complications [41]. Initially, when an injury occurs, the body triggers an inflammatory response to fight infection and remove damaged tissue. Pro-inflammatory cytokines like IL-6 and TNF-α are crucial in this phase, as they recruit immune cells to the site of injury [42]. However, if inflammation persists too long, it can lead to chronic inflammation, which can delay healing and cause further tissue damage. EVs, at lower concentrations, reduce levels of IL-6 and TNF-α, suggesting that they help to resolve inflammation at an appropriate time, preventing it from becoming chronic. This anti-inflammatory action creates an environment that is conducive to healing [43]. Other studies suggest a similar hypothesis whereby exosomes derived from various stem cells and other sources effectively reduce levels of pro-inflammatory IL-6 and TNF-α [44,45]. This reduction is mediated through the delivery of specific microRNAs that target inflammatory pathways [46,47].

On the other hand, cytokines like IL-33 and TGF-β are linked to tissue regeneration. Their increase after EV treatment suggests a shift toward healing and repair processes [48]. TGF-β, for example, plays a key role in collagen production and scar formation [49], while IL-33 can activate other pathways, such as the PI3K/AKT/mTOR and Wnt/β-catenin signaling pathways, that encourage tissue repair [50]. By promoting these cytokines, EVs help to transition the wound from the inflammatory phase to the regenerative phase, facilitating tissue rebuilding and remodeling. One study also mentioned that EVs derived from bovine colostrum contain anti-inflammatory factors that help to transition wounds from the inflammatory to the proliferative phase, promoting re-epithelialization, angiogenesis, and ECM maturation [51]. Thus, the dual role of EVs in mitigating harmful inflammation while enhancing repair mechanisms highlights their potential as a promising therapeutic approach for wound healing.

The robust increase in growth factors, including VEGF, BDNF, KGF, and IGF, particularly at an EV concentration of 50 μg/mL, underscores the pro-angiogenic and neurotrophic effects of UC-MSC-EVs. These findings highlight the potential of EVs to not only stimulate wound healing, but also improve the vascularization and structural integrity of regenerating tissues. Supporting this, other studies have shown that UC-MSC-derived exosomes enhance the delivery and effectiveness of these growth factors, thereby facilitating tissue regeneration and repair [52]. VEGF plays a crucial role in promoting the growth of new blood vessels, which are critical for supplying oxygen and nutrients to reparative cells. Additionally, VEGF also impacts the action of non-endothelial cells, such as keratinocytes and macrophages, in wound healing. It promotes keratinocyte proliferation, survival, and re-epithelialization through VEGFR-1, with its inhibition leading to delayed healing. In macrophages, VEGF recruits M2-polarized macrophages, which contribute to an anti-inflammatory environment and support tissue repair. These macrophages release pro-angiogenic factors, enhancing vascularization and endothelial cell growth, which are crucial for effective wound healing [53,54]. KGF is known to stimulate epithelial cell differentiation and proliferation while increasing levels of IGF-I and collagen IV. The combination of IGF-I with KGF accelerates both re-epithelialization and neovascularization [55]. Moreover, exosomes are rich in various growth factors that significantly enhance wound healing, including fibroblast growth factor 2 (FGF-2) for fibroblast proliferation, hepatocyte growth factor (HGF) for cell migration and regeneration, and TGF-β for collagen synthesis and remodeling. Together, these factors facilitate a comprehensive approach to accelerating tissue repair and regeneration [56]. Notably, exosomes derived from MSCs that are encapsulated in hydrogels have been shown to significantly enhance wound closure and granulation tissue regeneration [57]. Overall, the synergistic effects of these growth factors, particularly when delivered through advanced systems like exosome-encapsulated hydrogels, hold great promise for improving wound healing outcomes.

ECM proteins are essential components of tissue architecture, providing the scaffolding necessary for cellular attachment, migration, and proliferation during wound healing [58]. The increased expression of these proteins suggests that UC-MSC-EVs play a crucial role in enhancing the formation of a robust and functional ECM, which is vital for effective tissue repair and regeneration. Collagens contribute to tensile strength, with type III collagen, which is predominant in the early healing stages, gradually being replaced by type I collagen as the healing process advances. Additionally, elevated levels of fibronectin promote cell adhesion and migration, guiding cells to the wound site and facilitating their proliferation [59]. Importantly, the optimal effects of EVs observed at a concentration range of 50–75 μg/mL indicate a therapeutic window within which regenerative effects are maximized, minimizing the risk of excessive oxidative stress or inflammation. Within this concentration range, EVs likely release an optimal quantity of growth factors that stimulate fibroblast proliferation, migration, angiogenesis, and ECM synthesis, without overwhelming the local tissue environment. Understanding the influence of EVs on ECM protein expression and identifying their therapeutic concentration range are crucial for developing effective wound healing therapies [60]. This knowledge allows for targeted delivery of growth factors and signaling molecules that promote robust healing outcomes in various wound types, including chronic and complex wounds. Overall, the upregulation of ECM proteins by UC-MSC-EVs represents a critical mechanism through which these vesicles enhance tissue remodeling and repair, underscoring their potential as promising tools in regenerative medicine. Supporting our findings, EVs derived from MSCs are rich in bioactive molecules that enhance fibroblast proliferation, stimulate angiogenesis, and support tissue regeneration, while simultaneously reducing inflammation [61,62]. Moreover, exosomes derived from DFs in a 3D culture model have been shown to carry proteins that are essential for ECM formation and remodeling, promoting cellular migration and proliferation through pathways influenced by cytokines such as IL-6 [63]. Exosomes derived from tendon stem cells have demonstrated significant potential in enhancing tendon healing by effectively balancing the synthesis and degradation of the ECM. This property underscores their therapeutic promise in tissue repair, indicating a valuable approach for treating tendon injuries [64].

**Figure 12:**
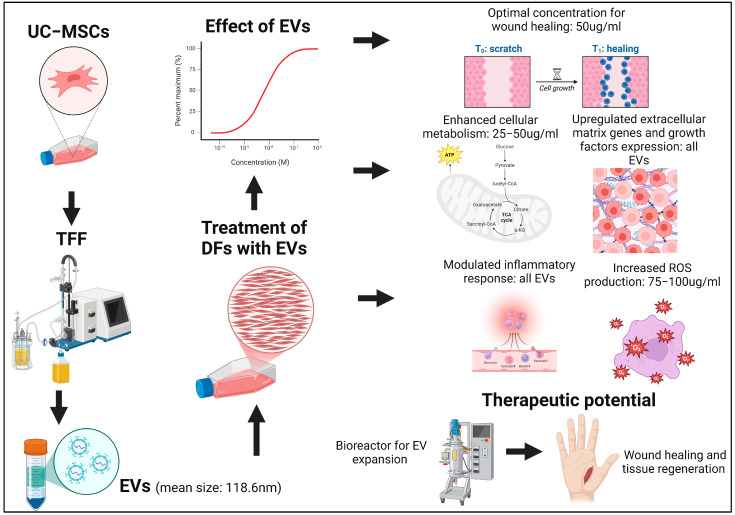
UC-MSC- EVs exhibit significant potential as cell-free therapeutic agents for wound healing, by modulating dermal fibroblast function through concentration-dependent effects. At the optimal concentration (50 μg/mL), EVs enhance metabolic activity, mitochondrial function, and ECM-related gene expression, while promoting balanced inflammatory responses and upregulating growth factors like VEGF, BDNF, and KGF. These findings highlight their multifaceted mechanisms of action, and emphasize the importance of dosing optimization for effective tissue regeneration in regenerative medicine.

The findings of this study highlight UC-MSC-EVs as promising therapeutic agents in regenerative medicine, particularly for dermal wound healing and tissue engineering. Their ability to modulate various aspects of DF function, including metabolic activity, migration, inflammation, growth factor production, and ECM synthesis, positions them as ideal candidates for treating chronic wounds or injuries where healing processes are impaired. Figure 12 summarizes the comprehensive analysis of the effects of UC-MSC-EVs on DF function and their wound healing applications. However, careful dose optimization is critical to fully harness their therapeutic potential, as excessive EV concentrations could diminish benefits through feedback mechanisms or excessive ROS production, underscoring the need for further research to establish optimal dosing strategies for effective clinical applications.

Future research on UC-MSC-EVs should focus on elucidating the specific molecular mechanisms underlying their therapeutic effects on DFs and other key cell types in wound healing. In vivo studies are essential to validate their efficacy in complex biological environments and to assess long-term safety and outcomes. Optimizing advanced delivery systems, such as hydrogels, will enhance the stability and targeted release of EVs, while exploring personalized medicine approaches can improve treatment efficacy based on individual patient responses. Additionally, addressing the heterogeneity of EVs, concentration-dependent effects, and the multifaceted nature of wound healing is crucial for developing standardized protocols for clinical application. While there are significant opportunities for expanding the therapeutic potential of UC-MSC-EVs in regenerative medicine, challenges related to regulatory considerations, funding, and the need for robust clinical trials must be carefully navigated in order to facilitate their successful translation into clinical practice.

## 5. Conclusions

This study provides compelling evidence for the therapeutic potential of UC-MSC-EVs in dermal wound healing and tissue regeneration. Lower concentrations of EVs (25–50 µg/mL) significantly enhanced metabolic and mitochondrial activity in DFs, indicating improved cellular energetics, while higher concentrations increased ROS levels, suggesting a potential hormetic effect that may contribute to adaptive responses. Additionally, EVs demonstrated anti-inflammatory effects by reducing pro-inflammatory cytokines (IL-6, TNF-α) and increasing pro-regenerative cytokines (IL-33, TGF-β). Treatment with EVs, particularly at 50 µg/mL, significantly elevated critical growth factors (VEGF, BDNF, KGF, IGF), promoting angiogenesis and tissue repair. Furthermore, EVs upregulated the expression of key ECM proteins (type I and III collagen, fibronectin) that are essential for wound healing and tissue remodeling. These findings underscore the multifaceted effects of UC-MSC-EVs on DF function, highlighting their potential as a novel therapeutic approach in regenerative medicine, with an emphasis on careful dose optimization to maximize therapeutic benefits while minimizing potential adverse effects.

## Figures and Tables

**Table 1:** Primer sequences used for real-time PCR.

Gene	Forward Primer (5′-3′)	Reverse Primer (5′-3′)
Type I collagen	AAGGAGGCCAGGTAGAAAGG	TGAGGATCAGGGGAAGGAGG
Type III collagen	GGGAGGAGGAGGAGGAGGAGG	TCACAGTGAGGAGAGGAGGA
Fibronectin	ATGGCGGAGTGGGATGGTGG	TGCTGTGGAGTGTGACAGTGG
Glyceraldehyde 3-phosphate dehydrogenase	CTGAGGAGCAGGGGGGAGAA	AAGGTCGGAGTCAACGGATTT

**Table 2:** The key findings from the NTA. The table consolidates the main data from the analysis, including particle size distribution statistics, concentration, and system settings during capture and analysis.

Parameter	Value
Mean Particle Size	119.2 ± 2.0 nm
Mode Particle Size	81.5 ± 2.8 nm
Standard Deviation (SD)	55.1 ± 2.6 nm
D10 (10% of particles below)	72.5 ± 1.7 nm
D50 (Median particle size)	101.1 ± 2.2 nm
D90 (90% of particles below)	184.8 ± 4.9 nm
Particle Concentration	1.83 × 10^12^ ± 1.66 × 10^11^ particles/mL
Particles per Frame	100.0 ± 9.1
Centers Detected per Frame	114.8 ± 10.8

**Table 3:** Summary of morphological changes and cell density in DFs exposed to varying concentrations of EVs.

Concentration of EVs (µg/mL)	Morphological Features	Cell Density	Observations
0 (Control)	Elongated, spindle-shaped morphology, with a well-spread appearance.	Normal	Typical fibroblast morphology observed in untreated cells.
25	Similar elongated, spindle-shaped morphology to control cells.	Slight increase	Cell density shows a minor increase compared to untreated cells, with no noticeable morphological changes.
50	Denser population, with enhanced spindle-like and healthier appearance.	Significant increase	Optimal concentration, resulting in reduced intercellular space and enhanced cell proliferation.
75	Retains fibroblast-like elongated morphology.	Comparable to 50 µg/mL	No further improvement in cell density or morphology compared to 50 µg/mL.
100	Similar fibroblast-like morphology as at 75 µg/mL.	Comparable to 50 µg/mL	No additional enhancement in morphology or cell density.

## Data Availability

The data supporting the reported results in this study will be made publicly available upon publication.
